# Social determinants of child abuse: Evidence from seven countries in sub-Saharan Africa

**DOI:** 10.1371/journal.pone.0305778

**Published:** 2024-07-17

**Authors:** Kwamena Sekyi Dickson, Edward Kwabena Ameyaw, Kenneth Setorwu Adde, Jones Arkoh Paintsil, Sanni Yaya

**Affiliations:** 1 Department of Population and Health, University of Cape Coast, Cape Coast, Ghana; 2 Institute of Policy Studies and School of Graduate Studies, Lingnan University, Tuen Mun, Hong Kong; 3 L & E; E Research Consult Ltd, Wa, Upper West Region, Ghana; 4 Department of Economics, Howard University, Washington DC, United States of America; 5 The George Institute for Global Health, Imperial College London, London, United Kingdom; COMSATS University Islamabad - Lahore Campus, PAKISTAN

## Abstract

**Background:**

Child abuse is a dominant public health concern that permeates race, varied social contexts and culture. Child abuse comprises any act of omission or commission perpetrated by a child’s parent, caregiver, or other adult leading to harm, potential for, or any threat of harm to a child (below age 18), either intentional or unintentional. This subject has usually been explored by focusing on men. This study investigated the prevalence and social correlates of child abuse across seven countries in sub-Saharan Africa.

**Methods and materials:**

Data was obtained from Demographic and Health Surveys (DHS) conducted in seven countries in sub-Saharan Africa between 2013 and 2020. The outcome variable employed for this study was acts of child abuse (including shouting, striking, and slapping). Descriptive and inferential analyses were carried out. The descriptive analysis focused on the bivariate analysis between the country variable and the outcome variables. Multivariate analysis was, however, utilized to determine the relationship between the outcome variables and the respondents’ explanatory variables, using a binary logistic regression model. The adjusted odds ratios for each variable were calculated using a 95% confidence range.

**Results:**

The proportion of women shouting at children was 72 percent. This ranged from 49.2 percent in Chad to 84.2 percent in Benin. The proportion of women striking children was 52.5 percent and this ranged from 37.1 percent in Chad to 63.8 percent in Benin. The odds of women striking their children was higher for those with children aged 10–14 (aOR = 1.18, CI = 1.03, 1.34), women with primary education (aOR = 1.25, CI = 1.17, 1.33), cohabiting women (aOR = 1.17, CI = 1.10, 1.25) and women who had experienced intimate partner violence (aOR = 1.06, CI = 1.00, 1.12). The odds of women shouting at their children was higher for those aged 30–34 years (aOR = 1.31, CI = 1.11, 1.55) and for working women (aOR = 1.43, 1.33, 1.56). The odds of women slapping their children was higher for those who justify wife-beating (aOR = 1.10, CI = 1.03, 1.16) and for women with richest wealth status (aOR = 1.25, CI = 1.17, 1.33).

**Conclusions:**

The findings show that it is imperative for the governments of the countries studied, especially those with high prevalence like Benin, to consider parent-friendly and culturally acceptable non-formal educational initiatives that will dissuade parents and guardians from abusing children. Possibly, legal reforms that sanction harsher punishments to perpetrators of child abuse may help make child abuse less attractive to parents and guardians.

## Introduction

Child abuse is a dominant public health concern and is common in almost all human settings irrespective of race, social context or culture [1, 2]. Child abuse is “any action, behavior and gestures by another person adult or child that causes considerable damage to a child. It can be physical, sexual or emotional, but can just as often be about a lack of affection, care and attention.” [3]. Acts of child abuse include shouting at children and striking or slapping [4]. The World Health Organization (WHO) acknowledges that child abuse has adverse consequences on a child’s survival, health, holistic development and dignity in the aspects of power, trust and responsibility [4]. Hence there are continuing efforts to end all forms of child abuse to secure a conducive environment for children’s development and well-being [5–7]. For instance, target 16.2 of the Sustainable Development Goals (SDGs) strives to “end abuse, exploitation, trafficking and all forms of violence against, and torture of, children” [8]. Abuse of children appears widespread across sub-Saharan Africa (SSA), despite the limited literature and statistics quantifying the magnitude [9]. Surveys have revealed that most children in SSA experience diverse violent disciplinary acts as almost one in three children are hit or beaten since age 15, whilst one in ten is raped or abused sexually [10].

Social determinants of health (SDH) have also been identified to affect children’s susceptibility to abuse [1]. SHD refers to the “conditions in which people are born, grow, live, work and age, including the health system” [11]. These factors determine the wellbeing, quality of life and freedom of individuals [12]. This is shaped by the circulation of wealth as well as resources and power at the local, national and global levels [11, 12]. The social conditions that perpetrate child abuse need to be identified to inform appropriate policies and interventions.

Victims of child abuse persistently experience poorer physical and mental health outcomes compared with other children [13–15]. Children who suffer abuse have high risks of HIV infection, risk of re-victimization and bullying [16, 17]. Considering that children require a healthy safe nurturing and responsive living environment [18], there is a need for an empirical study to comprehend the specific social determinants that affect children’s health and their prospects of being abused.

Several factors have been linked to child abuse. Previous studies from elsewhere have reported that factors that induce child abuse include poverty or low wealth status, being lower than three years, female sex, and children in families with three or more children [19, 20]. In Egypt, a cross-sectional study also revealed that factors such as the younger age of mothers, poverty, urban residence and having 3–5 children contribute to child abuse [1]. In China, some identified underlying factors of child abuse include the socio-economic status of the child’s family, maternal education, and occupational circumstances [21]. Additional factors include the physical health status of the child, disability, and the caregiver-child relationship [22]. Relatedly, in a Nepal-based study where nearly half of the children had experienced moderate physical abuse, it was evident that higher education and high household wealth status shielded children from being abused [23]. Similar reports have emerged from other parts of the world [24–26]. All these factors culminate into the social determinants of children’s health. A Zimbabwean study also noticed that some parents/caregivers may be unaware of the differences between disciplining a child and abusing a child [27], suggesting that some parents/caregivers may abuse children ignorantly.

Considering the paucity of empirical evidence on the prevalence and social correlates of child abuse in SSA, this study investigates the social determinants of child abuse. Considering that much scholarly attention has been on abuse perpetrated by men/males thereby portraying child abuse as masculine to a greater extent [28, 29], the present study strives to offer a divergent perspective by focusing on women alone. It is envisaged that the outcome of this study will direct pragmatic anti-child abuse initiatives required to protect the fundamental human rights of children and offer them a conducive atmosphere that will facilitate their holistic growth and development to their full potential.

## Methods

### Data source

We obtained data from seven countries in sub-Saharan Africa. Countries were included if they had their latest Demographic and Health Surveys (DHS) between 2013 and 2020. Secondly, the dataset set of each of the countries must include variables that are of interest to our study. Using a two-stage stratified cluster sampling approach, the surveys chose nationally representative samples of women of reproductive age (15–49 years). The DHS is excellent for our study because it captures detailed data on maternal (antenatal care, delivery, and postnatal care), child (nutrition, abuse), fertility, family planning, infant and child mortality, and other areas. The study included women with a birth history who had given birth up to five years before the survey. The data for the study was collected from mothers. Following a review of our concept note, the MEASURE DHS granted permission to use the data set. The datasets are available to the public for free at The DHS Program, and available here: https://www.dhsprogram.com/data/available-datasets.cfm

### Study variables and measurements

#### Outcome variable

The study had three main outcomes, which were acts of child abuse by mothers. Mothers’ actions of child abuse were measured using replies to questions about whether they had committed the following acts of child abuse against their children in the last month before the survey: 1. Shouted at their children (no, yes); 2. Struck or hit their children (no, yes); 3. Slapped their children (no, yes) [1]. Hence the outcomes are shouting at children, striking children and slapping children.

#### Explanatory variables

Thirteen explanatory variables were used by both theoretical and empirical literature [1, 30, 31]. These include Child variables [sex of the child (male, female), age of the child (0–4, 5–9, 10–14, 15–17)], maternal variables [number of children (0–2, 3–5, 6+) women’s age (15–19, 20–24, 25–29, 30–34, 35–39, 40–44, 45–49 years), place of residence (urban, rural), level of education (no education, primary, secondary, higher) wealth quintile (poorest, poorer, middle, richer, richest), occupation (not working, working)], and violence variables [intimate partner violence (IPV) (no, yes), justifies wife-beating (no, yes), experience of childhood violence] (no, yes). Country comprized Benin, Burundi, Congo DR, Liberia, Chad, Togo and Uganda.

Justification of wife-beating was a composite variable derived from 1. neglect of a child; 2. burning of food; 3. arguing with husband/partner; 4. refusal to have sex with husband/partner; 5. going out without permission. All these were asked as yes = 1 or no = 0. An index was created with all the “yes” and “no” answers, with scores ranging from 0 to 5. The score 0 was labelled as “no”, and 1 to 5 was labelled as yes.

Intimate partner violence was a composite variable derived from the following: 1. experience of any sexual violence by husband/partner; 2. Experience any emotional violence by husband/partner; and 3. Experience any physical violence by husband/partner. All these were asked as yes = 1 or no = 0. An index was created with all the “yes” and “no” answers, with scores ranging from 0 to 3. The score 0 was labelled as “no”, and 1 to 3 was labelled as yes. Generational violence was created from response to the question “ever physically hurt by father” and “ever physically hurt by mother”.

### Statistical analyses

Descriptive and inferential analyses were carried out. The descriptive analysis looked at that frequency, and the proportions of the background characteristics by the outcome variables. Multivariate analysis (made of adjusted and unadjusted models) was then utilized to determine the relationship between the outcome variables and the respondents’ explanatory variables, using a binary logistic regression model. Three hierarchical models were fit. Model I examined the relationship between the child variables [sex of child, age of child] and the outcome variables. Model II examines the associations of child variables [sex of child, age of child] and maternal variable [number of children, women’s age, place of residence, level of education, wealth status, current marital status, Occupation] and the outcome variables. Model III is a complete model that examined all the explanatory variables [sex of child, age of child, number of children, women’s age, place of residence, level of education, wealth status, current marital status, Occupation, intimate partner violence, justifies wife-beating, experience of childhood violence, country variable] and the outcome variables.

A binary logistic regression model was used based on the dichotomous nature of the outcome variables. Each variable was subjected to a multicollinearity test, which revealed a mean-variance inflation factor (VIF) of 2.66 for the variables in the models. According to Chatterjee [32], a VIF score of higher than 10 indicates the presence of multicollinearity. The adjusted odds ratios for each variable were calculated using a 95% confidence range. Stata was used to handle and analyze the data (version 17). To account for any under- or over-sampling in the entire sample, the results were sample-weighted.

### Ethical considerations

This study used de-identified, secondary data gathered as part of the DHS program. We did not obtain ethical clearance for the conduct of this study since the data is publicly available for its usage. However, permission for use was sought from the MEASURE DHS and approval was granted before downloading and utilizing it for the study. Before the commencement of the survey, ethical approval was obtained, and all ethical criteria governing the use of human participants were strictly adhered to. http://goo.gl/ny8T6X contains information about data and ethical standards.

## Results

### Descriptive results

The proportion of women shouting at children was 72 percent. This ranged from 49.2 percent in Chad to 84.2 percent in Benin. The proportion of women striking children was 52.5 percent and this ranged from 37.1 percent in Chad to 63.8 percent in Benin. Also, the proportion of women slapping children was 33.3 percent. This ranged from 17.9 percent in Togo to 46.4 percent in Burundi (see Table 1).

**Table 1:** Country, year of survey, by child abuse among women.

Country	Year of survey	N	Shouted at children (95% Cl)	Struck children (95% Cl)	Slapped children (95% Cl)
Benin	2017–2018	2,537	84.2 (83.7, 84. 6)	63.8 (63.2, 64.4)	46.2 (45.6, 46.9)
Burundi	2016–2017	4,579	83.7 (83.3, 84.0)	49.6 (49.2, 50.1)	46.4 (45.9, 46.9)
Congo DR	2013–2014	1,556	60.2 (59.4, 60.9)	49.3 (48.5, 50.1)	42.5 (41.7, 43.3)
Liberia	2019–2020	1,195	83.2 (82.5, 83.9)	54.3 (53.4, 55.2)	34.9 (34.0, 35.8)
Chad	2014–2015	1,642	49.4 (48.6, 50.1)	37.1 (36.4, 37.9)	21.8 (21.2, 22.4)
Togo	2013–2014	3,169	70.2 (69.7, 70.7)	43.1 (42.6, 43.7)	17.9 (17.5, 18.4)
Uganda	2016	5,014	66.2 (65.8, 66.6)	61.1 (60.6, 61.5)	25.0 (24.6, 25.4)
Total		19,692	72.4 (72.2, 72.)	52.5 (52.3, 52.7)	33.3(33.1, 33.5)

Women with female children (72.5%), children aged 15–17 years (72.6%), women aged 30–34 years (73.4%), from urban residence (73.5%), with no education (73.7%), with richest wealth status (72.8%), working (73.4%), who had experience intimate partner violence (72.9%), who does not justify wife-beating (74.2%) and have no experience of generational violence (72.5%) had the highest proportion of shouting at children (see Table 2).

**Table 2:** Background characteristics and prevalence of child abuse.

Background Characteristics	N	Shouted at children (95% Cl)	Struck children (95% Cl)	Slapped children (95% Cl)
***Sex of child***				
Male	9,995	72.2 (72.0, 72.5)	52.1 51.8, 52.4)	33.4 (33.1, 33.7)
Female	9,697	72.5 (72.2, 72.8)	52.9 (52.6, 53.2)	33.2 (32.9, 33.5)
***Age of child***				
0–4	15,558	72.4 (72.2, 72.7)	52.3 (52.1, 52.6)	33.6 (33.4, 33.8)
5–9	2,951	72.2 (71.6, 72.6)	52.7 (52.1, 53.3)	32.3 (31.8, 32.9)
10–14	948	71.9 (71.0, 72.8)	56.6 (55.5, 57.5)	33.2 (32.3, 34.2)
15–17	235	72.6 (70.7, 74.3)	46.9 (44.9, 49.0)	25.6 (23.9, 27.4)
***Number of children***				
0–2	6,217	73.2 (72.8, 73.5)	52.6 (52.2, 53.0)	33.5 (33.1, 33.9)
3–5	8,385	72.9 (72.6, 73.2)	52.3 (52.0, 52.7)	33.2 (32.9, 33.5)
6+	5,090	70.5 (70.1, 70.8)	52.8 (52.3, 53.2)	33.2 (32.8, 33.6)
***Women’s age***				
15–19	710	69.3 (68.2, 70.4)	54.9 (53.8, 56.1)	35.1 (34.0, 36.2)
20–24	3,367	71.0 (70.5, 71.5)	52.7 (52.2, 53.2)	32.6 (32.1, 33.1)
25–29	4,628	72.6 (72.2, 73.0)	51.6 (51.1, 52.0)	33.2 (32.8, 33.1)
30–34	4,086	73.4 (73.0, 73.9)	51.9 (51.4, 52.4)	34.0 (33.6, 34.5)
35–39	3,222	72.7 (72.2, 73.2)	53.0 (52.4, 53.5)	32.9 (32.4, 33.4)
40–44	2,147	72.1 (71.5, 72.7)	53.8 (53.1, 54.4)	32.9 (32.2, 33.6)
45–49	1,522	72.9 (72.1, 73.6)	52.9 (52.1, 53.7)	33.6 (32.9, 34.4)
***Place of residence***				
Urban	5,188	73.5 (73.1, 73.8)	54.3 (53.9, 54.8)	32.9 (32.5, 33.3)
Rural	14,504	72.0 (71.7, 72.2)	51.9 (51.6, 52.1)	33.4 (33.2, 33.7)
***Level of education***				
No education	7,789	73.7 (73.4, 73.9)	50.4 (50.0, 50.7)	35.9 (35.6, 36.2)
Primary	7,856	72.3 (72.0, 72.6)	54.7 (54.4, 55.1)	31.1 (30.7, 31.4)
Secondary	3,530	69.9 (69.4, 70.4)	52.5 (52.0, 53.0)	33.2 (32.7, 33.7)
Higher	517	70.7 (69.4, 71.9)	52.3 (51.0, 53.7)	28.7 (27.5, 30.0)
***Wealth status***				
Poorest	4,185	71.5 (71.0, 71.9)	51.6 (51.1, 52.1)	33.8 (33.3, 34.2)
Poorer	4,119	72.6 (72.1, 73.0)	53.2 (52.7, 53.7)	34.4 (33.8, 34.8)
Middle	4,002	72.4 (72.0, 72.9)	52.5 (52.0, 53.0)	32.2 (31.7, 32.6)
Richer	3,792	72.7 (72.2, 73.1)	52.7 (52.2, 53.2)	32.0 (31.5, 32.5)
Richest	3,594	72.8 (72.2, 73.2)	52.7 (52.2, 53.2)	34.1 (33.6, 34.6)
***Current marital status***				
Married	12,070	72.1 (71.9, 72.3)	51.8 (51.5, 52.1)	33.9 (33.6, 34.2)
Cohabitation	5,384	72.8 (72.3, 73.1)	53.7 (53.3, 54.2)	31.7 (31.3, 32.1)
Widowed	707	75.0 (74.0, 76.0)	54.1 (52.9, 55.2)	33.9 (32.8, 35.0)
Divorced	220	70.0 (68.0, 71.9)	47.2 (45.1, 49.3)	39.2 (37.1, 41.2)
Separated	1,311	72.0 (68.0, 71.9)	54.5 (53.7, 55.4)	32.7 (31.9, 41.2)
***Occupation***				
Not working	3,081	66.6 (66.1, 67.1)	49.5 (48.9, 50.0)	30.3 (29.8, 30.8)
Working	16,611	73.4 (73.2, 73.7)	53.1 (52.8, 53.3)	33.9 (33.6, 34.1)
***Intimate Partner Violence (IPV***)				
No	10,316	71.9 (71.6, 72.1)	51.9 (51.6, 52.2)	33.1 (32.8, 33.4)
Yes	9,375	72.9 (72.6, 73.2)	53.2 (52.9, 53.5)	33.5 (33.2, 33.8)
***Justifies wife beating***				
No	9,785	74.2 (73.9, 74.4)	53.9 (53.6, 54.2)	32.3 (32.0, 32.6)
Yes	9,907	70.6 (70.3, 70.9)	51.2 (50.9, 51.5)	34.2 (33.9, 34.5)
***Generational violence***				
No	18,416	72.5 (72.2, 72.7)	52.7 (52.5, 52.9)	33.5 (33.3, 33.7)
Yes	1,276	71.2 (70.4, 71.9)	50.1 (49.3, 51.0)	30.3 (29.5, 31.1)
Total	19,692	72.4 (72.2, 72.)	52.5 (52.3, 52.7)	33.3 (33.1, 33.5)

Furthermore, women aged 15–19 years (54.9%), with female children (52.9%), children aged 10–14 years (56.6%), from urban residence (54.3%), with primary education (54.7%), with poorer status (53.2%), working (53.1%), who had experience intimate partner violence (53.2%), who does not justify wife-beating (53.9%) and have no experience of generational violence (52.7%) had the highest proportion of striking children (see Table 2).

Also, Women with male children (33.4%), children aged 0–4 years (33.6%), with 0–2 children (33.5%) women aged 15–19 years (35.1%), from rural residence (33.4%), with no education (35.9%), with poorer wealth status (34.4%), working (33.9%), who had experienced intimate partner violence (33.5%), who justify wife-beating (34.2%) and have no experience of generational violence (33.5%) had the highest proportion of slapping children (see Table 2).

### Inferential analysis results

The odds of women shouting at their children was lower for those with 6+ children (aOR = 0.90, CI = 0.83, 0.98) compared to women with 0–4 children. The odds of women shouting at their children was 1.31 times higher for those aged 30–34 (aOR = 1.31, CI = 1.11, 1.55) and 1.43 times higher for women who were working (aOR = 1.43, 1.33, 1.56) compared to those aged 15–19 and those not working respectively (see Table 3). The odds of women shouting at their children was lower for those in the rural residence (aOR = 0.88, CI = 0.82, 0.94) compared to those in the urban residence. Also, the odds of women shouting at their children was 1.18 times higher for women with the richest wealth status and 1.12 times higher for cohabiting women compared to women with poorest wealth status and married women respectively (see Table 3). The odds of women shouting at their children was lower for women who justified wife-beating (aOR = 0.87, CI = 0.81, 0.92) and Chad women (aOR = 0.17, 0.14, 0.19) compared to those who did not justify wife-beating and Benin women respectively (see Table 3).

**Table 3:** Binary logistic regression on socio-demographic characteristics and child abuse (Shouted at children).

Background Characteristics	Shouted at children Adjusted Odds Ratio (95% Confidence level)	Shouted at children Adjusted Odds Ratio (95% Confidence level)	Shouted at children Adjusted Odds Ratio (95% Confidence level)
	Model I	Model II	Model III
***Sex of child***			
Male	Ref	Ref	Ref
Female	1.03(0.97, 1.10)	1.03(0.97, 1.09)	1.03(0.97, 1.10)
***Age of child***			
0–4	Ref	Ref	Ref
5–9	1.01(0.92, 1.10)	0.90(0.82, 1.00)	1.01(0.92, 1.10)
10–14	0.98(0.8, 1.14)	0.82**(0.69, 0.96)	0.98(0.85, 1.14)
15–17	1.01(0.75, 1.35)	0.79(0.8, 1.08)	1.01(0.75, 1.35)
***Number of children***			
0–2		Ref	Ref
3–5		0.86**(0.79, 0.95)	1.01(0.93, 1.08)
6+		0.71***(0.63, 0.81)	0.90*(0.83, 0.98)
***Women’s age***			
15–19		Ref	Ref
20–24		1.17(0.98, 1.39)	1.17(0.98, 1.39)
25–29		1.30**(1.09, 1.56)	1.24**(1.04, 1.46)
30–34		1.47***(1.21, 1.77)	1.31**(1.11, 1.55)
35–39		1.51***(1.24, 1.85)	1.27**(1.06, 1.51)
40–44		1.56***(1.26, 1.94)	1.25*(1.04, 1.50)
45–49		1.73***(1.36, 2.21)	1.28*(1.06, 1.55)
***Place of residence***			
Urban		Ref	Ref
Rural		0.88**(0.81, 0.97)	0.88***(0.82, 0.94)
***Level of education***			
No education		Ref	Ref
Primary		0.95(0.89, 1.03)	1.01(0.94, 1.08)
Secondary		0.84***(0.7, 0.92)	0.94(0.86, 1.03)
Higher		0.73**(0.58, 0.92)	0.93(0.75, 1.16)
***Wealth status***			
Poorest		Ref	Ref
Poorer		1.08(0.99, 1.19)	1.08(0.98, 1.18)
Middle		1.11*(1.01, 1.22)	1.09(0.99, 1.20)
Richer		1.09(0.99, 1.21)	1.09(1.07, 1.31)
Richest		1.17**(1.03, 1.32)	1.18**(1.07, 1.31)
***Current marital status***			
Married		Ref	Ref
Cohabitation		1.14**(1.0, 1.23)	1.12**(1.04, 1.21)
Widowed		1.08(0.91, 1.29)	1.11(0.93, 1.31)
Divorced		0.86(0.64, 1.14)	0.89(0.67, 1.18)
Separated		1.03(0.91, 1.18)	1.05(0.93, 1.20)
***Occupation***			
Not working		Ref	Ref
Working		1.43***(1.32, 1.56)	1.43***(1.33, 1.56)
***Intimate Partner Violence (IPV***)			
No			Ref
Yes			1.05(0.99, 1.12)
***Justifies wife beating***			
No			Ref
Yes			0.87***(0.81, 0.92)
***Generational violence***			
No			Ref
Yes			0.89(0.79, 1.01)
***Country***			
Benin			Ref
Burundi			0.94(0.82, 1.07)
Congo DR			0.27***(0.24, 0.32)
Liberia			0.86(0.72, 1.02)
Chad			0.17***(0.14, 0.19)
Togo			0.43***(0.38, 0.49)
Uganda			0.39***(0.35, 0.44)

*p<0.05

**p<0.0

***p<0.001 Ref = Reference category

The odds of women striking their children was 1.18 times higher for those with children aged 10–14 years (aOR = 1.18, CI = 1.03, 1.34) and 1.25 times higher for women with primary education (aOR = 1.25, CI = 1.17, 1.33) compared to those with children aged 0–4 years and with no education respectively. Also, the odds of women striking children was 1.17 time higher for cohabiting women (aOR = 1.17, CI = 1.10, 1.25) and 1.06 times higher for women who had experienced intimate partner violence (aOR = 1.06, CI = 1.00, 1.12) compared to married women and those had not experienced intimate partner violence (see Table 3). The odds of women striking their children was lower for those from rural residences (OR = 0.91, CI = 0.85, 0.97) compared to those in urban residences (see Table 4).

**Table 4:** Binary logistic regression on socio-demographic characteristics and child abuse (Struck children).

Background Characteristics	Struck children Adjusted Odds Ratio (95% Confidence level)	Struck children Adjusted Odds Ratio (95% Confidence level)	Struck children Adjusted Odds Ratio (95% Confidence level)
	Model I	Model II	Model III
***Sex of child***			
Male	Ref	Ref	Ref
Female	1.04(0.99, 1.10)		1.04(0.99, 1.18)
***Age of child***			
0–4	Ref	Ref	Ref
5–9	1.00(0.92, 1.08)	0.98(0.89, 1.07)	1.00(0.92, 1.08)
10–14	1.17**(1.03, 1.34)	1.13(0.98, 1.32)	1.18*(1.03, 1.34)
15–17	0.80(0.62, 1.05)	0.77(0.59, 1.02)	0.80(0.62, 1.04)
***Number of children***			
0–2		Ref	Ref
3–5		1.02(0.94, 1.10)	0.98(0.91, 1.04)
6+		1.02	0.97(0.90, 1.04)
***Women’s age***			
15–19		Ref	Ref
20–24		1.00(0.85, 1.17)	1.00(0.86, 1.18)
25–29		0.91(0.77, 1.07)	0.91(0.78, 1.06)
30–34		0.95(0.80, 1.13)	0.94(0.81, 1.10)
35–39		0.98(0.81, 1.18)	0.96(0.82, 1.13)
40–44		0.99(0.81, 1.21)	0.98(0.83, 1.16)
45–49		1.00(0.81, 1.25)	0.96(0.81, 1.15)
***Place of residence***			
Urban		Ref	Ref
Rural		0.87**(0.80, 0.94)	0.91**(0.85, 0.97)
***Level of education***			
No education		Ref	Ref
Primary		1.22***(1.14, 1.30)	1.25***(1.17, 1.33)
Secondary		1.13**(1.03, 1.23)	1.15**(1.06, 1.25)
Higher		1.16(0.94, 1.43)	1.16(0.96, 1.41)
***Wealth status***			
Poorest		Ref	Ref
Poorer		1.07(0.99, 1.17)	1.08(0.99, 1.17)
Middle		1.05(0.96, 1.14)	1.07(0.98, 1.16)
Richer		1.00(0.91, 1.09)	1.05(0.96, 1.15)
Richest		0.95(0.85, 1.06)	1.06(0.97, 1.16)
***Current marital status***			
Married		Ref	Ref
Cohabitation		1.14***(1.07, 1.22)	1.17***(1.10, 1.25)
Widowed		1.01(0.87, 1.18)	1.04(0.90, 1.21)
Divorced		0.92(0.70, 1.20)	0.95(0.73, 1.24)
Separated		1.15*(1.02, 1.29)	1.20**(1.06, 1.35)
***Occupation***			
Not working		Ref	Ref
Working		1.20***(1.11, 1.30)	0.97(0.87, 1.09)
***Intimate Partner Violence (IPV***)			
No			Ref
Yes			1.06**(1.00, 1.12)
***Justifies wife beating***			
No			Ref
Yes			0.91**(0.86, 0.97)
***Generational violence***			
No			Ref
Yes			0.97(0.87, 1.09)
***Country***			
Benin			Ref
Burundi			0.56***(0.51, 0.62)
Congo DR			0.54***(0.48, 0.62)
Liberia			0.66***(0.58, 0.76)
Chad			0.31***(0.28, 0.36)
Togo			0.42***(0.37, 0.46)
Uganda			0.94(0.85, 1.04)

*p<0.05

**p<0.01

***p<0.001 Ref = Reference category

The odds of women slapping their children was 1.10 times higher for those who justify wife-beating (aOR = 1.10, CI = 1.03, 1.16) and 1.11 times higher for women with richest wealth status (aOR = 1.25, CI = 1.17, 1.33) compared to those who do not justify wife-beating and with poorest wealth status respectively. Moreover, the odds of women slapping children was lower for those with higher education (aOR = 0.74, CI = 0.60, 0.92) and for women who had their children aged 15–17 (aOR = 0.74, CI = 0.55, 0.99) compared to women with no education and those had aged 0–4 (see Table 5).

**Table 5:** Binary logistic regression on socio-demographic characteristics and child abuse (Slapped children).

Background Characteristics	Slapped children Adjusted Odds Ratio (95% Confidence level)	Slapped children Adjusted Odds Ratio (95% Confidence level)	Slapped children Adjusted Odds Ratio (95% Confidence level)
	Model I	Model II	Model III
***Sex of child***			
Male	Ref	Ref	Ref
Female	1.02(0.96, 1.08)	1.01(0.96, 1.08)	1.02(0.98, 1.08)
***Age of child***			
0–4	Ref	Ref	Ref
5–9	0.94(0.86, 1.02)	0.88**(0.80, 0.97)	0.94(0.86, 1.02)
10–14	1.01(0.88, 1.17)	0.92(0.79, 1.08)	1.02(0.88, 1.17)
15–17	0.74*(0.55, 0.99)	0.65**(0.48, 0.89)	0.74*(0.55, 0.99)
***Number of children***			
0–2		Ref	Ref
3–5		0.92(0.85, 1.00)	0.98(0.91, 1.05)
6+		0.87**(0.78, 0.98)	0.96(0.89, 1.04)
***Women’s age***			
15–19		Ref	Ref
20–24		0.92(0.78, 1.10)	0.94(0.79, 1.11)
25–29		0.95(0.80, 1.14)	0.95(0.81, 1.12)
30–34		1.04(0.86, 1.25)	1.02(0.86, 1.20)
35–39		1.00(0.82, 1.21)	0.95(0.80, 1.23)
40–44		1.07(0.87, 1.32)	0.99(0.84, 1.18)
45–49		1.10(0.87, 1.38)	0.98(0.81, 1.18)
***Place of residence***			
Urban		Ref	Ref
Rural		0.97(0.90, 1.06)	0.95(0.88, 1.01)
***Level of education***			
No education		Ref	Ref
Primary		0.85***(0.79, 0.91)	0.87***(0.82, 0.93)
Secondary		0.93(0.85, 1.02)	0.99(0.91, 1.08)
Higher		0.64***(0.51, 0.80)	0.74**(0.60, 0.92)
***Wealth status***			
Poorest		Ref	Ref
Poorer		1.04(0.95, 1.13)	1.02(0.94, 1.12)
Middle		1.00(0.92, 1.10)	0.98(0.90, 1.07)
Richer		0.99(0.89, 1.09)	0.96(0.88, 1.06)
Richest		1.16**(1.03, 1.30)	1.11*(1.01, 1.22)
***Current marital status***			
Married		Ref	Ref
Cohabitation		0.98(0.91, 1.05)	0.97(0.90, 1.03)
Widowed		1.00(0.85, 1.18)	0.99(0.84, 1.16)
Divorced		1.19(0.90, 1.57)	1.17(0.89, 1.54)
Separated		0.98(0.86, 1.11)	0.96, 0.85, 1.09)
***Occupation***			
Not working		Ref	Ref
Working		1.25***(1.15, 1.36)	1.22***(1.12, 1.32)
***Intimate Partner Violence (IPV***)			
No			Ref
Yes			1.01(0.96. 1.08)
***Justifies wife beating***			
No			Ref
Yes			1.10**(1.03, 1.16)
***Generational violence***			
No			Ref
Yes			0.89(0.79, 1.00)
***Country***			
Benin			Ref
Burundi			0.98(0.89, 1.09)
Congo DR			0.81**(0.71, 0.92)
Liberia			0.59***(0.52, 0.68)
Chad			0.30***(0.26, 0.35)
Togo			0.25***(0.22, 0.28)
Uganda			0.41***(0.37, 0.45)

*p<0.05

**p<0.01

***p<0.001 Ref = Reference category

## Discussion

This study investigated the prevalence and social correlates of child abuse across seven countries in sub-Saharan Africa. Three main indicators were used; shouting at children, striking children and slapping children. Averagely, 72%, 52.5% and 33.3% of children experienced shouting, striking and slapping correspondingly. Nationally, children from Benin experienced the highest prevalence of shouting (84.2%) and striking (63.8%), meanwhile, Burundi children recorded the highest prevalence of slapping (46.4%). The lowest prevalence of shouting (49.2%) and striking (37.1%) occurred in Chad, however, Togo had the least share of slapping (17.9%). These findings highlight the varied nature of child abuse and the potential role of contextual factors in shaping the specific abuse a child is likely to suffer depending on his/her country, society or context of origin [4, 33]. These findings suggest that adapting anti-child abuse intervention ditto might not be effective, hence the need for well-thought-through and fit-for-purpose child protection interventions at local, national and sub-regional levels [34, 35].

The odds of shouting at children was lower among rural residents compared to urban residents. Similarly, the odds of women striking their children was lower for those in rural locations. Rurality is intertwined with strong family and social cohesion, less stress from traffic and lower work demands [36]. These could strengthen parental bonding and familial empathy towards one another and thereby reduce the chances of abusing one’s child. The recounted characteristics of rural lifestyle seem to shield rural children from abuse of all kinds, compared with their counterparts in densely populated urban locations, whose parents will spend hours in traffic and return home with work-related stress and on the verge of anger of the least provocation [37, 38]. Besides, urbanization and its associated pressures truncate children’s protection against violence, abuse and neglect [39].

The odds of striking was higher for children 10–14 years compared to those aged 0–4. There are different forms of punishing or disciplining children and considering that children between 0–4 years are tender compared to those in the 10–14 age range, the finding is expected. Thus, it is expected that the older a child, the more likely the child will be punished through a relatively harsher corporal punishment like striking. However, some scholars have argued that parents who consider spanking as socially unacceptable are not successful in developing their children’s behavior to mature into responsible adults [40]. Disruptive behaviours in early childhood (e.g., aggressive, oppositional, and hyperactive conduct) usually lead to predictive adverse mental health outcomes in later life, and manifest in school failure, substance abuse and other unacceptable acts [9]. The government of Benin, where shouting and striking dominated, needs to consider educational interventions that will re-orient parents to appreciate that even though it is rewarding to have socially upright children, they should be cautious about the punishments they administer to the children in their early stages of life, in order not to abuse them. In the case of Benin, where most cases of child abuse emerged, our findings were expected the country is notable for various forms of child abuse [41]. Aside from the types of abuse we investigated, a significant proportion of children in Benin suffer from child labour and exploitation of different kinds [41]. Consequently, an expert from the United Nations has decried the alarming nature of child abuse in the country. The Benin government might have to intensify the implementation strategies of existing laws on child abuse.

The odds of striking was higher for children of cohabiting women compared to children of married women. A number of factors may account for this finding. Throughout Africa, childbearing has much social recognition when it occurs within marriage, suggesting some level of prestige and social protection enjoyed by children born within marriages as compared to children who are products of cohabitation or other unrecognized unions [42]. Besides, a greater proportion of women in cohabitation suffer varied forms of abuse compared to the married [43]. One pathway is that several women in cohabitation are expectant that their partners will marry them and hence would not like to do anything that will mar their chances of being married [44]. Due to this, some of them endure abusive partners and as such have limited negotiation power to protect their children from abuse by their partners and by so doing the women may welcome the act as a way of securing their relationships [45]. Consequently, some scholars have questioned whether cohabitation is a license for abuse [46].

These factors could account for why children of cohabiting women had a higher odds of being struck as compared to the children of married women. Women empowerment initiatives that propel females to appreciate human dignity and autonomy, may be instrumental to salvage the situation. Children of women with IPV experience had a higher odds of being struck. This corroborates findings by other studies from different parts of the world as several studies have reported the same [1, 47, 48]. In Egypt, Seleem and Amer [49] also noted that abused parents have higher chances of disciplining their children through violent methods. This finding plausibly implies the inability of abused mothers to respond appropriately to children’s misconduct possibly as a result of the consequences of the stress they have experienced from IPV victimization. It may also reflect abusive mothers’ approach of stopping a child from arousing the anger of an abusive partner, thereby increasing the risk of the mother and her partner abusing the child [1, 31].

The study interestingly revealed that women who justify wife beating had lesser odds of striking children. This finding appears inconsistent with some existing evidence on women’s disposition to wife beating and its association with child abuse [50, 51]. Similarly, evidence from Somalia indicates that women with favourable disposition towards wife beating supported child maltreatment in the form of psychological aggression and moderate to severe physical assault of any kind [52]. In spite of the divergence, our finding could be that the women consider children to be more vulnerable and hence require much protection unlike adult women who can fend for themselves or initiate any legal action against anyone who abuses them. This seemingly protective notion might be rooted in gender construction which characterised mothers are carers of children [53].

In consonance with the existing body of knowledge [52, 53], justification of wife beating was associated with an increased tendency of slapping children. When juxtaposed with the disposition of these women towards child striking, we can infer that women who justify wife beating have preference for how a child is to be abused, thus choosing slapping over striking. These findings highlight the critical need for interventions on child abuse to take into account women’s disposition towards wife-beating, and thereby highlight the possible adverse implications of the method utilised to abuse a child (e.g. slapping or striking).

On slapping, children with a higher odds of being slapped had the following characteristics: had mothers who justify wife beating or belonged to rich mothers. Meanwhile, the odds of slapping was lower among children whose mothers had higher education and for women who had children aged 15–17. Education exposes women to fundamental human rights and the need to protect the wellbeing of the feeble such as children and a plethora of evidence have already established that maternal education is protective for children [54–56]. The findings therefore reinforce the importance of female education to be prioritized in the countries studied, as this serves as a protective tool against child abuse.

Considering that both wife-beating and slapping of children are abusive, it is not surprising that children whose mothers justify wife-beating had an increased odds of being slapped. Realizing higher odds of slapping among children of richer women may be due to several factors. For instance, it could be that these women are very busy with economic activities and hence leave the children in the care of others [57]. This can increase the chances that such children will suffer abuse, including slapping. Considering the depth of relevant information education instills [58], it is expected that children whose mothers have received higher education will have decreased odds of being slapped.

The study showed that relative to Benin, the tendency to strike, slap or shout at children was lower in all other countries. These interesting results possibly imply that all the three modalities of abusing children are very prevalent in Benin relative to the other countries. It is worth mentioning that Togo, Chad and DR Congo recorded the lowest tendencies of slapping, striking and shouting correspondingly. The finding has unraveled the commonest forms of abuse that children in these countries are less likely to encounter relative to children in Benin. This is not a guarantee that children in these countries do not risk any form of abuse. Considering the variation in modalities of child abuse, interventions on this subject must be well thought through in order and be well-tailored to meet the contextual circumstances driving specific modalities in the different countries.

### Strengths and limitations

Despite the extensive documentation of child abuse in African countries [30, 58], only a little empirical evidence on the social factors that reinforce this act exists. Using appropriate analytical procedures, this study extends the frontier of knowledge on child abuse by unravelling the prevalence and social correlates in seven SSA countries. The methodological approach support generalisation of our findings and recommendations to all children in the studied countries. Despite the acknowledged strengths, causal inference between the examined social determinants and child abuse is impossible due to the cross-sectional nature of the study. Secondly, though child abuse is a broad concept, we only focused on shouting, striking, and slapping considering that these were the only variables available in the dataset we used. Thirdly, considering that child abuse is unwelcome in all human settings, it is possible that the findings might have been influenced by social desirability bias. Meanwhile, the study provides a true reflection of child abuse within the confines of the three outcomes measured.

## Conclusions

The study has shown that, of the seven countries studied, children in Benin experience the highest burden of shouting and striking. Meanwhile, slapping was predominant among Burundi children. We noted that the least prevalence of shouting and striking in Chad whilst the lowest burden of slapping occurred in Togo. The study showed that women who live in rural areas are less prone than those who live in urban areas to yell at their children. While cultural background (Chad women) and rationalizing wife-beating are linked to decreased odds of screaming, socioeconomic factors including wealth position and cohabitation are associated with increased probabilities of shouting. These findings imply that residency, social status, views on violence, and cultural background are strongly linked to how women behave towards their children. The findings have shown that generic interventions cannot mitigate child abuse across SSA, as abuse manifests in diverse ways across nationalities and settings. It is, therefore, imperative that governments of the countries studied, especially Benin and Burundi, consider parent-friendly and culturally acceptable non-formal educational initiatives that will dissuade parents and guardians from abusing children. Possibly, legal reforms that sanction harsher punishments to perpetrators of child abuse may be helpful in making child abuse less attractive to parents and guardians. Through these measures, a conducive environment can be created for the holistic development of children, in order for them to be assertive enough and develop to their full potential. The identified risk factors of each of the three child abuse indicators (e.g. absence of formal education in the case of child slapping) ought to guide existing policies on child abuse and subsequent legislative instruments that will be developed to avert child abuse in the countries studied. Future studies on this subject could adapt a qualitative lens to unravel why a mother would use a particular modality of child abuse over several other options or modalities. Intervention studies involving counselling and better parenting skills may also be helpful to increase mothers’ awareness about available legal actions against child abuse and better ways to raise children devoid of abuse.

## Supporting information

S1 File PLoS inclusivity in global research questionnaire. (DOCX)

S2 File PLoS human subjects research checklist. (DOCX)

S3 File STROBE checklist. (DOCX)
